# SFRP2 drives aerobic glycolysis and tumor progression in ovarian cancer by transcriptional upregulation of PTK2B

**DOI:** 10.1186/s12967-026-08480-9

**Published:** 2026-06-23

**Authors:** Jindong Sheng, Yiwen Xing, Jing Luan, Xiangyu Liu, Hualin Song, Lu Sun, Min Yu, Xin Fu, Huijuan Wu, Ying Chen, Wenxin Liu, Ke Wang

**Affiliations:** 1 Department of Gynaecological Oncology, Tianjin Medical University Cancer Institute and Hospital, Tianjin, China; 2 National Clinical Research Center for Cancer, Key Laboratory of Cancer Prevention and Therapy of Tianjin, Tianjin’s Clinical Research Center for Cancer, Tianjin, China; 3 Department of Pathology, The Second Hospital of Tianjin Medical University, Tianjin, China

**Keywords:** Ovarian cancer, SFRP2, PTK2B, Aerobic glycolysis, Transcriptional regulation

## Abstract

**Background:**

Ovarian cancer is a highly lethal gynecologic malignancy, with metabolic reprogramming being a key contributor to its progression and therapeutic resistance. Although Secreted Frizzled-Related Protein 2 (SFRP2) has been implicated in various cancers, its functional role and molecular mechanisms in ovarian cancer, particularly in regulating metabolic pathways, remain poorly defined.

**Methods:**

Bioinformatic analyses of GEO (GSE66957) and TCGA-OV datasets were performed to assess SFRP2 expression and its prognostic correlation. Immunohistochemistry (IHC) on a human ovarian cancer tissue microarray was used for clinical validation. SFRP2 was knocked down or overexpressed in ovarian cancer cell lines (HEY and SK-OV-3) using lentiviral shRNA vectors. Functional assays (CCK-8, colony formation, apoptosis, migration) and metabolic assays (glucose consumption, lactate/ATP production, ECAR/OCR) were conducted. The mechanistic link between SFRP2, transcription factor CEBPA, and downstream target PTK2B was investigated using co-immunoprecipitation (Co-IP), nuclear-cytoplasmic fractionation, chromatin immunoprecipitation (ChIP), and dual-luciferase reporter assays. Rescue experiments were performed both in vitro and in vivo (xenograft mouse models).

**Results:**

SFRP2 was significantly overexpressed in ovarian cancer tissues and cell lines, and high SFRP2 expression correlated with advanced disease and poor patient survival. SFRP2 knockdown suppressed cell proliferation, colony formation, and migration, while promoting apoptosis. Mechanistically, SFRP2 interacted with the transcription factor CEBPA, promoted its nuclear translocation, and enhanced its occupancy at the PTK2B promoter, thereby driving PTK2B transcriptional activation. This SFRP2/CEBPA/PTK2B axis further activates the AKT/mTOR signaling pathway, upregulating key glycolytic enzymes. Importantly, inhibiting glycolysis with 2-DG or knocking down PTK2B effectively reversed the oncogenic effects of SFRP2 both in vitro and in vivo.

**Conclusion:**

Our findings identify a novel SFRP2/CEBPA/PTK2B signaling axis that drives aerobic glycolysis and malignant progression in ovarian cancer, highlighting SFRP2 and PTK2B as potential prognostic markers and therapeutic targets.

**Supplementary Information:**

The online version contains supplementary material available at https://doi.org/10.1186/s12967-026-08480-9.

## Introduction

Ovarian cancer is the most lethal gynecological malignancy, with approximately 80% of patients presenting with stage III-IV disease and widespread peritoneal dissemination. Although most initially respond to platinum-based chemotherapy, > 85% relapse because of intrinsic or acquired chemoresistance, resulting in a 5-year survival rate of less than 25% and underscoring the urgent need for new therapeutic strategies [1, 2]. A hallmark of cancer cells, including ovarian cancer, is metabolic reprogramming, particularly the Warburg effect or aerobic glycolysis. Under aerobic conditions, cancer cells preferentially metabolize glucose to lactate even in the presence of oxygen [3]. This metabolic shift provides cells with the necessary biomass and energy to support rapid proliferation, invasion, and resistance to apoptosis. Given the critical role of metabolic reprogramming in ovarian cancer pathogenesis and treatment resistance, clarifying its upstream regulators and downstream effectors is essential [4].

SFRP2 (Secreted Frizzled-Related Protein 2), a member of the secreted frizzled-related protein family, is a secreted modulator of Wnt signaling that can inhibit canonical Wnt/β-catenin or activate non-canonical Wnt/Ca²⁺ pathways in a context-dependent manner [5–7]. In cancer, SFRP2 often behaves as a “double-edged sword” but predominantly exerts tumor-promoting effects by stimulating angiogenesis, metabolic reprogramming and metastasis, and has emerged as a potential therapeutic target [8, 5, 9]. In ovarian cancer, SFRP2 has been identified as an independent poor prognostic marker [10]. Its overexpression may promote disease progression by activating the Wnt/β-catenin pathway (e.g., through interaction with the miR-96-5p/MTSS1 axis [11]) or by cooperating with other oncogenic factors such as ARPC1B [12]. Additionally, SFRP2 is involved in processes such as fibrotic transformation [11] and chemotherapy resistance [13]. However, the precise function and mechanism of SFRP2 in ovarian cancer progression, particularly its impact on aerobic glycolysis and malignant phenotypes, are largely unknown.

PTK2B (Protein Tyrosine Kinase 2 Beta), a non-receptor tyrosine kinase and member of the Focal Adhesion Kinase (FAK) family, regulates cell adhesion, migration and survival and has been implicated in synaptic plasticity and tumor progression [14, 15]. In ovarian cancer, emerging evidence implicates PTK2B in disease progression through multiple pathways. Notably, PTK2B/FAK inhibitors such as PF-431,396 have been shown to reverse resistant cell phenotypes and act synergistically with chemotherapy, suggesting potential for overcoming platinum resistance [14]. Additionally, PTK2B may influence the peritoneal metastatic microenvironment—where adhesion and migration are critical—by modulating tumor cell-microenvironment interactions through LPXN phosphorylation [14, 16]. Furthermore, while PTK2B overexpression has been linked to stem cell maintenance in leukemia, analogous mechanisms could potentially sustain stemness properties in ovarian cancer stem cells (OCSCs) [14, 17]. However, its regulation and functional contribution to ovarian cancer metabolism, particularly in glycolysis, are unknown.

In this study, we initially identified SFRP2 as a significantly overexpressed gene correlated with poor survival in ovarian cancer. We systematically demonstrate that SFRP2 acts as a potent oncoprotein that drives malignant progression by enhancing aerobic glycolysis. Furthermore, we elucidate a novel transcriptional mechanism whereby SFRP2 interacts with the transcription factor CCAAT/Enhancer-Binding Protein Alpha (CEBPA) to activate PTK2B expression. Our findings establish the SFRP2/CEBPA/PTK2B axis as a critical regulator of glycolytic metabolism and tumor progression in ovarian cancer, revealing new prognostic and therapeutic opportunities.

## Materials and methods

Core experimental procedures (qPCR, Western blotting, Co-IP, metabolic assays, ChIP, luciferase assays, and xenograft models) are described in the main text, whereas supporting assays and extended protocols are provided in the Supplementary Information.

### Bioinformatic analysis

SFRP2 expression data were downloaded from the GEO database (GSE66957). Survival analysis based on SFRP2 expression was performed using RNA-seq and clinical data from the TCGA-OV project accessed via the GDC portal. Gene Set Enrichment Analysis (GSEA) was conducted using GSEA software (v4.3.2) with the Hallmark gene sets.

### Tissue microarray and IHC

Ovarian cancer and adjacent normal tissues from a commercial TMA (Shanghai Outdo Biotech, Cat. No. HOvaC181SU01) were used for IHC staining of SFRP2. Ethical approval was obtained from the Medical Ethics Committee of Tianjin Medical University Cancer Institute and Hospital (Approval No. Ek2022065), and informed consent was provided. The TMA cohort consisted of 100 ovarian cancer tissues and 81 para-carcinoma tissues. After excluding the detached or damaged spots (10 tumor cases and 14 para-carcinoma cases), a total of 90 tumor and 67 para-carcinoma spots were evaluable for SFRP2 expression analysis. IHC staining was conducted following the standard protocol. Briefly, TMA slides were deparaffinized in xylene, rehydrated through graded ethanol, and subjected to antigen retrieval in citrate buffer (pH 6.0). Endogenous peroxidase was blocked with 3% H₂O₂, followed by incubation with 5% normal goat serum. Anti-SFRP2 antibody (Wuhan Sanying, Cat. No. 66328-1-Ig; 1:200) was applied overnight at 4 °C, then detected with HRP-conjugated secondary antibody (Proteintech, 1:500) and DAB chromogen. Hematoxylin was used for counterstaining. Staining intensity (0–3) and positive cell percentage (0–4) were scored, with the final IRS (0–12) categorized as low (0–3), moderate (4–6), or high (7–12) expression.

### Cell culture and transfection

Human ovarian cancer cell lines (HEY, SK-OV-3, HO-8910) and the normal ovarian epithelial cell line IOSE80 were obtained from the American Type Culture Collection (ATCC, Manassas, VA, USA) and cultured in RPMI-1640 medium (Gibco, Thermo Fisher Scientific, USA; Cat. No. 11875119) supplemented with 10% fetal bovine serum (FBS; Gibco; Cat. No. 10099141) and 1% penicillin-streptomycin (Gibco; Cat. No. 15140122) at 37 °C in a humidified incubator with 5% CO₂. For lentiviral transduction, cells were seeded at 50–60% confluence and infected with lentiviral vectors carrying shRNAs targeting SFRP2 or PTK2B (GeneChem Co., Ltd., Shanghai, China), or overexpression plasmids for SFRP2 (GeneChem), in the presence of 5 µg/mL polybrene (Sigma-Aldrich, USA; Cat. No. TR-1003-G). Stable cell lines were selected using puromycin (2 µg/mL; InvivoGen, USA; Cat. No. ant-pr-1) for 7–10 days. The glycolysis inhibitor 2-deoxy-D-glucose (2-DG; Sigma-Aldrich; Cat. No. D8375) was dissolved in sterile PBS and used at a final concentration of 10 mM in complete culture medium. Target sequences for shRNAs against SFRP2 and PTK2B are provided in Table S1. All experiments were performed with cells at 70–80% confluence and within 15 passages to ensure consistency.

### Quantitative real-time PCR (qPCR) assay

Total RNA was extracted using TRIzol and reverse-transcribed into cDNA. qPCR was performed using SYBR Green on a CFX96 system, and relative expression was calculated by the 2^−ΔΔCt^ method using GAPDH as control. Primer sequences are provided in Supplementary Table S2.

### Western blotting (WB) assay

Protein lysates were prepared in RIPA buffer and analyzed by SDS-PAGE followed by transfer to PVDF membranes. After incubation with primary and HRP-secondary antibodies, signals were visualized using ECL. Antibody information is listed in Supplementary Table S3.

### Metabolic assays

#### Glucose consumption, lactate production, and ATP levels

Intracellular glucose consumption, lactate production, and ATP levels were quantified using specialized assay kits purchased from Solarbio Science & Technology (Beijing, China). Specifically, glucose levels were measured using the Glucose Content Assay Kit (Cat. No. BC2500), lactate production was assessed with the Lactate Content Assay Kit (Cat. No. BC2230), and intracellular ATP levels were determined using the ATP Content Assay Kit (Cat. No. BC0300). All experimental procedures were strictly performed following the manufacturer’s instructions. To ensure accuracy, the metabolic data were normalized to the total protein concentration of each sample, which was determined using a BCA Protein Assay Kit (Beyotime, Cat. No. P0012).

### Extracellular flux analysis (ECAR and OCR)

Real-time measurements of the Oxygen Consumption Rate (OCR) and Extracellular Acidification Rate (ECAR) were conducted using a Seahorse XF96 Extracellular Flux Analyzer (Agilent Technologies, Santa Clara, CA, USA). For the Mitochondrial Stress Test (OCR): Cells were seeded in XF96 cell culture microplates. After baseline measurement, the following compounds were sequentially injected: Oligomycin (1.5 µM) to inhibit ATP synthase, FCCP (1.0 µM) to uncouple oxidative phosphorylation and induce maximal respiration, and Antimycin A (0.5 µM) to shut down mitochondrial respiration. For the Glycolysis Stress Test (ECAR): Cells were equilibrated in glucose-free Seahorse base medium. The assay involved sequential injections of Glucose (10 mM) to trigger glycolysis, Oligomycin (1.5 µM) to shift energy production to maximum glycolysis, and 2-deoxyglucose (2-DG, 50 mM) to inhibit glycolysis and measure non-glycolytic acidification. All data were recorded in real-time and normalized to either cell number or total protein content per well to account for variations in cell density. Data analysis was performed using Wave software (Agilent).

### Co-immunoprecipitation (Co-IP)

To investigate the interaction between SFRP2 (Proteintech, 55309-1-AP) and CEBPA (OmnimAbs, OM284260), Co-IP was performed. Cells were lysed in RIPA buffer with protease inhibitors. Cell lysates (500 µg) were incubated with 5 µg anti-SFRP2 antibody overnight at 4 °C, followed by 20 µL protein A/G beads (Thermo, 88802) for 2 h. Normal rabbit IgG was used as a negative control. Beads were washed, and bound proteins were eluted with SDS loading buffer. Inputs and immunoprecipitates were analyzed by Western blot using anti-SFRP2 and anti-CEBPA antibodies. This method confirmed the potential interaction between SFRP2 and CEBPA.

### Chromatin immunoprecipitation (ChIP) assay

ChIP was performed to analyze CEBPA binding to the PTK2B promoter using a SimpleChIP^®^ Kit (Cell Signaling, Cat. No. 9003). Cells were cross-linked with 1% formaldehyde, and chromatin was fragmented by sonication. Chromatin (5 µg) was immunoprecipitated overnight at 4 °C with 5 µg anti-CEBPA antibody (OmnimAbs, Cat. No. OM284260) or normal rabbit IgG (negative control). Immune complexes were captured with Protein G magnetic beads, washed, and eluted. Cross-links were reversed, and DNA was purified. PTK2B promoter enrichment was quantified by qPCR using primers targeting the predicted CEBPA-binding site (sequences in Table S2). Binding levels were calculated as % input. IgG controls confirmed specificity.

### Dual-luciferase reporter assay

The interaction between CEBPA and the PTK2B promoter was assessed using the dual-luciferase system (Promega, Cat. No. E1910). The PTK2B promoter fragment was cloned into pGL3-basic (Firefly luciferase), with pRL-TK (Renilla luciferase) as an internal control. HEY and SK-OV-3 cells were co-transfected with pGL3-PTK2B-Luc, pRL-TK, and either pcDNA3.1-CEBPA (overexpression) or siCEBPA (knockdown). Firefly luciferase activity (normalized to Renilla) was measured 48 h post-transfection using a luminometer. Relative luciferase activity (%) reflected CEBPA-dependent PTK2B promoter regulation. Triplicate wells and ≥ 3 biological replicates ensured reproducibility.

### In vivo xenograft model

Female nude mice (4–6 weeks old) were purchased from Jiangsu GemPharmatech Co., Ltd. (Nanjing, China). All animal procedures were approved by the Institutional Animal Care and Use Committee (IACUC) of the Animal Ethical and Welfare Committee of Tianjin Medical University Cancer Institute and Hospital (Approval No. [NSFC-AE-2022061]) and conducted in accordance with the Guide for the Care and Use of Laboratory Animals. For tumor xenografts, 5 × 10⁶ cells (resuspended in 100 µL PBS) were subcutaneously injected into the right flanks of mice (*n* = 6 per group). Experimental groups included: NC, SFRP2, shPTK2B, and SFRP2 + shPTK2B. Tumor size was measured every 3 days using calipers, and tumor volume (mm³) was calculated as: Volume = 0.5 × length × width². After 33 days, mice were euthanized by CO₂ asphyxiation followed by cervical dislocation, and tumors were excised, weighed, and photographed. Tumor tissues were either fixed in 4% paraformaldehyde for histological analysis or snap-frozen in liquid nitrogen for further molecular studies.

### Statistical analysis

Data are presented as mean ± SD. Differences between groups were analyzed using Student’s t-test (two groups) or one-way ANOVA (multiple groups). The association between SFRP2 expression and clinicopathological characteristics was analyzed using the Pearson’s chi-square test. Correlation between SFRP2 expression and tumor size was evaluated using Spearman’s rank correlation. Survival analysis was performed using the Kaplan-Meier method and Cox proportional hazards model. *P* < 0.05 was considered statistically significant.

## Results

### SFRP2 is overexpressed in ovarian cancer and correlates with poor prognosis

**Fig. 1:**
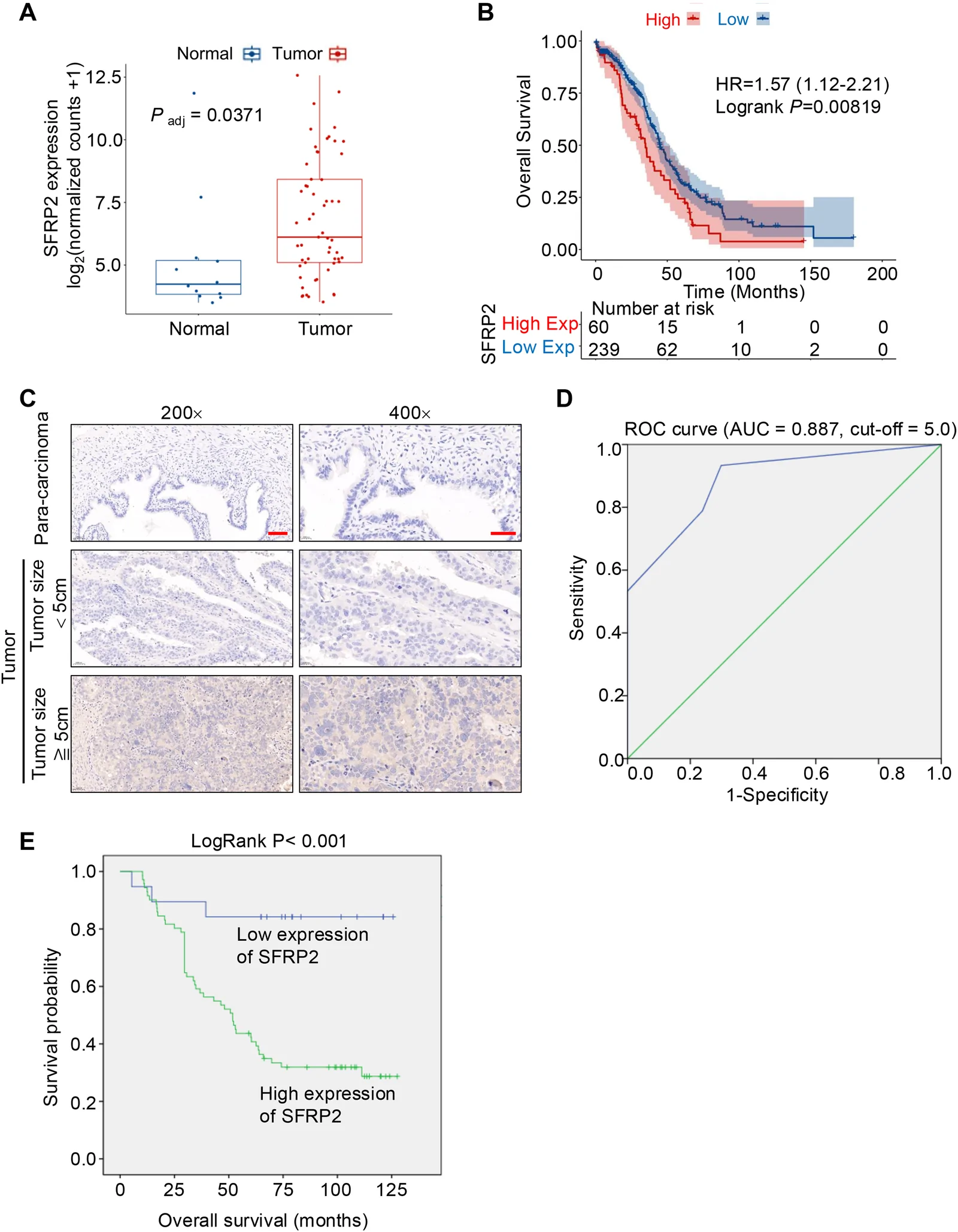
SFRP2 is highly expressed in ovarian cancer and correlates with poor prognosis. (**A**) Boxplot showing SFRP2 mRNA expression levels in ovarian cancer (Tumor) versus adjacent non-tumor tissues (Normal) based on RNA-seq data. Expression values are log₂-transformed and normalized (log₂ (normalized counts + 1)). Statistical significance was determined using DESeq2/Limma, with *P*_adj_=0.0371 and log_2_FC = 1.69. Each dot represents an individual sample. (**B**) Kaplan–Meier survival curve illustrating overall survival (OS) in patients stratified by SFRP2 expression level (high vs. low). High SFRP2 expression (*n* = 60) is associated with significantly shorter OS compared to low expression (*n* = 239). Hazard ratio (HR) = 1.57 (95% CI: 1.12–2.21), Log-rank *P* = 0.00819. Numbers at risk are indicated below the curve. (**C**) Representative immunohistochemical (IHC) staining of SFRP2 in ovarian cancer tissues and para-carcinoma. Staining intensity increases with advancing tumor stage. Images are shown at 200× and 400× magnification. Brown staining indicates positive SFRP2 expression. Scale bar = 50 μm. (**D**) Receiver operating characteristic (ROC) curve analysis of SFRP2 expression. ROC curve analysis was performed to determine the optimal cut-off value for SFRP2 expression based on the Immunoreactive Score (IRS). The area under the curve (AUC) was 0.887, and the optimal cut-off value was identified as 5.00 (maximum Youden index). Samples with an IRS > 5.0 were classified as the high-expression group, while those with an IRS ≤ 5.0 were classified as the low-expression group. (**E**) Kaplan–Meier survival analysis of overall survival in ovarian cancer patients grouped by SFRP2 protein expression (low vs. high) based on IHC results. Patients with high SFRP2 expression exhibit significantly worse survival outcomes (Log-rank *P* < 0.001)

To define the clinical relevance of SFRP2 in ovarian cancer, we first assessed its expression and prognostic value. Bioinformatic analyses of the GEO dataset (GSE66957) revealed that SFRP2 mRNA levels were significantly elevated in ovarian cancer tissues compared with normal ovarian tissues (Fig. 1A). Consistent with this, analysis of the TCGA-OV cohort demonstrated that high SFRP2 expression was associated with significantly shorter overall survival (OS) (Fig. 1B). We validated these findings at the protein level using immunohistochemistry (IHC) on a human ovarian cancer tissue microarray, which confirmed marked upregulation of SFRP2 in cancer tissues compared to para-carcinoma controls (Fig. 1C; Table 1). To objectively categorize SFRP2 expression, Receiver Operating Characteristic (ROC) curve analysis was performed, identifying an optimal immunoreactive score (IRS) cutoff value of 5.0 (AUC = 0.887), which was used to stratify patients into high- and low-expression groups (Fig. 1D). Correlation analysis between SFRP2 expression and clinicopathological parameters revealed that high SFRP2 expression was positively correlated with larger tumor size (Tables 2 and 3). Critically, Kaplan-Meier survival analysis of our patient cohort confirmed that high SFRP2 protein expression predicted a poorer prognosis (Fig. 1E). To further evaluate its prognostic value, we performed Cox proportional hazards analysis. Although SFRP2 expression was not identified as an independent prognostic factor in the multivariate model after adjusting for age, FIGO stage, and pathological grade (Table S4), its significant association with tumor progression and patient survival in our cohort firmly establishes SFRP2 as a clinically relevant oncoprotein in ovarian cancer.

**Table 1:** SFRP2 expression patterns in ovarian cancer tissues and para-carcinoma tissues revealed by immunohistochemical analysis

SFRP2 expression	Tumor tissue		Para-carcinoma tissue		*P* value
	Cases	Percentage	Cases	Percentage	
Low	19	21.1%	51	76.1%	*P *< 0.001
High	71	78.9%	16	23.9%	

**Table 2:** Relationship between SFRP2 expression and tumor characteristics in patients with ovarian cancer

Features	No. of patients	SFRP2 expression		*P* value
		low	high	
All patients	90	19	71	
Age				0.285
<53 years	43	7	36	
≥ 53 years	47	12	35	
Grade				0.290
non-HGSOC	5	2	3	
HGSOC	85	17	68	
Tumor size				0.028
<5 cm	46	14	32	
≥ 5 cm	44	5	39	
FIGO stage				0.396
I	13	3	10	
II	3	1	2	
III	69	15	54	
IV	5	0	5	

**Table 3:** Relationship between SFRP2 expression and tumor characteristics in patients with ovarian cancer

		SFRP2
Tumor size	Spearman	0.234
	Significance (two-tailed)	0.027
	N	90

### Knockdown of SFRP2 suppresses malignant phenotypes in vitro

**Fig. 2:**
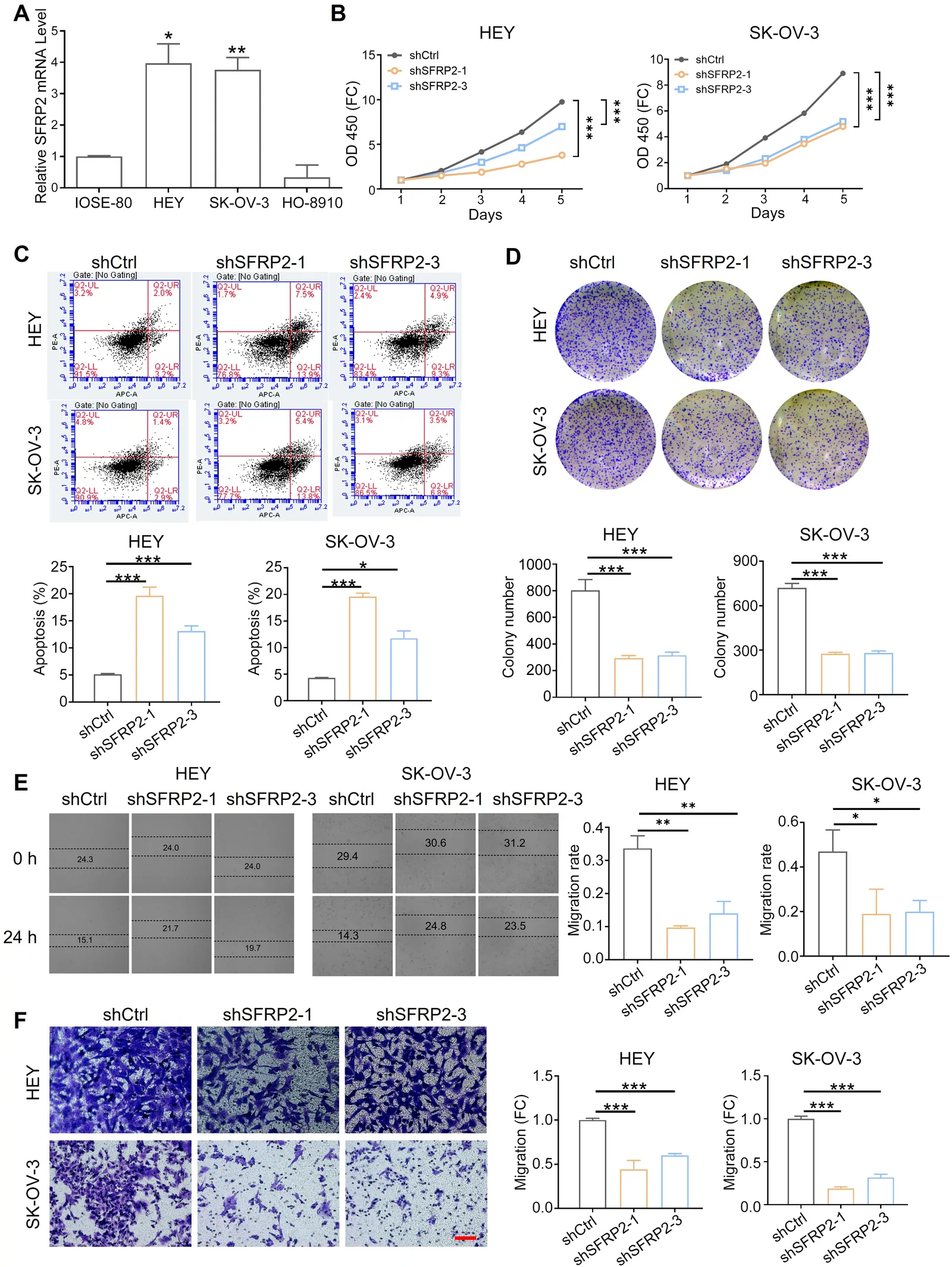
Knockdown of SFRP2 suppresses malignant phenotypes of ovarian cancer cells. (**A**) Quantitative real-time PCR analysis of SFRP2 mRNA expression in human ovarian epithelial cell line IOSE80 and ovarian cancer cell lines HEY, SK-OV-3 and HO-8910. Data are presented as mean ± SD (*n* = 3). *P < 0.05, **P < 0.01 vs. IOSE80. (**B**) Cell proliferation assays performed using CCK-8 kit in HEY and SK-OV-3 cells transfected with shCtrl, shSFRP2-1, or shSFRP2-3. Optical density (OD) at 450 nm was measured daily over 5 days. SFRP2 knockdown significantly reduced cell proliferation compared to control. **P < 0.01, ***P < 0.001. (**C**) Apoptosis analysis by flow cytometry using Annexin V-FITC/PI staining in HEY and SK-OV-3 cells after SFRP2 knockdown. Representative dot plots show early (Q4) and late (Q3) apoptotic populations. Bar graphs summarize percentage of apoptotic cells. SFRP2 silencing increased apoptosis in both cell lines. *P < 0.05, ***P < 0.001. (**D**) Colony formation assay assessing long-term proliferative capacity. Representative images and quantification of colonies formed by HEY and SK-OV-3 cells following SFRP2 knockdown. Knockdown of SFRP2 significantly reduced colony number. ***P < 0.001. (**E**) Wound healing assay evaluating cell migration in HEY and SK-OV-3 cells. Representative images at 0 and 24 h post-wounding. Migration rate was calculated as the ratio of wound closure at 24 h to initial wound width. SFRP2 depletion significantly impaired migratory ability. *P < 0.05, **P < 0.01, ***P < 0.001. (**F**) Transwell migration assay assessing invasive potential. Representative images of migrated cells stained with crystal violet (scale bar = 100 μm). Quantification shows fold change (FC) in migrated cell numbers relative to control. SFRP2 knockdown markedly reduced cell migration. ***P < 0.001

To further validate the oncogenic role of SFRP2 in ovarian cancer, we performed a series of in vitro functional assays. qPCR analysis confirmed that SFRP2 mRNA was highly expressed in ovarian cancer cell lines such as HEY, SK-OV-3 compared to the normal ovarian epithelial cell line IOSE80 (Fig. 2A). Based on these expression profiles, we selected HEY and SK-OV-3 cells for subsequent functional studies. To establish stable SFRP2-knockdown models, we designed three specific shRNAs targeting SFRP2. Transfection of these shRNAs into HEY and SK-OV-3 cells, followed by qPCR and western blot analyses, identified shSFRP2-#1 and shSFRP2-#3 as the most efficient sequences for SFRP2 knockdown (Figure S1A-C).

Functional assays demonstrated that SFRP2 knockdown significantly inhibited cell proliferation, as evidenced by CCK-8 assays (Fig. 2B). Flow cytometric analysis showed a significant increase in the apoptosis rate in SFRP2-knockdown cells compared with the negative control (shCtrl) (Fig. 2C). Furthermore, the clonogenic survival of SFRP2-knockdown cells was drastically impaired, as indicated by fewer and smaller colonies formed in the colony formation assay (Fig. 2D). Cell migration, assessed by both wound healing and Transwell assays, was also markedly suppressed in SFRP2-knockdown cells compared to controls (Fig. 2E and F). These results demonstrate that SFRP2 is essential for maintaining the proliferation, survival, and migratory capacity of ovarian cancer cells.

### SFRP2 drives tumor progression by enhancing aerobic glycolysis

**Fig. 3:**
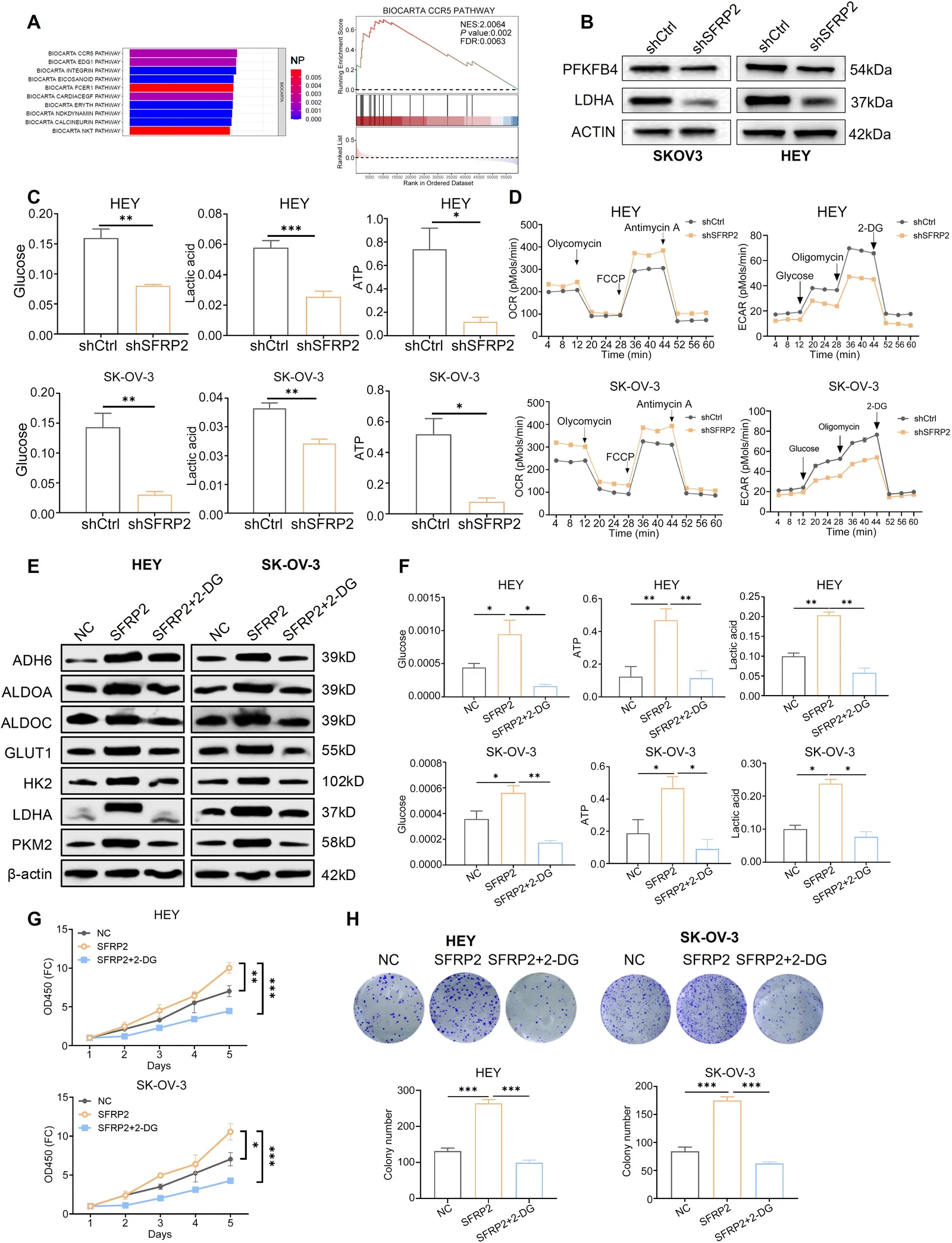
SFRP2 promotes ovarian cancer progression by enhancing aerobic glycolysis. (**A**) Gene set enrichment analysis (GSEA) of TCGA-OV transcriptomic data showing significant enrichment of the “HALLMARK_CCR5_SIGNALING” gene set in tumors with high SFRP2 expression (normalized enrichment score, NES = 2.18; false discovery rate, FDR q-value < 0.001). The leading-edge analysis heatmap displays expression of core-enriched genes in the CCR5 pathway. (**B**) Western blot analysis of key glycolytic proteins PFKFB4 and LDHA in SK-OV-3 and HEY cells following SFRP2 knockdown (shSFRP2) or control (shCtrl). Actin serves as loading control. SFRP2 silencing reduces protein expression of PFKFB4 and LDHA. (**C**) Metabolic assays measuring glucose consumption, lactate production, and ATP levels in HEY and SK-OV-3 cells after SFRP2 knockdown. Glucose uptake and lactate release are significantly reduced, while ATP levels are markedly decreased in shSFRP2 cells compared to controls. *P < 0.05, **P < 0.01, ***P < 0.001. (**D**) Real-time extracellular flux analysis assessing oxygen consumption rate (OCR) and extracellular acidification rate (ECAR) in HEY and SK-OV-3 cells. OCR reflects mitochondrial respiration, while ECAR indicates glycolytic activity. SFRP2 knockdown increases OCR (indicating enhanced oxidative phosphorylation) and decreases ECAR (indicating suppressed glycolysis), consistent with metabolic shift away from aerobic glycolysis. (**E**) Western blot analysis of glycolytic enzymes in HEY and SK-OV-3 cells treated with SFRP2 overexpression (SFRP2), SFRP2 + 2-DG (a glycolysis inhibitor), or negative control (NC). Expression of PKM2, HK2, GLUT1, ALDOC, ALDOA, ADH6, and LDHA is increased by SFRP2 overexpression but attenuated by 2-DG co-treatment. β-Actin serves as loading control. (**F**) Metabolic phenotyping of HEY and SK-OV-3 cells under SFRP2 overexpression or SFRP2 + 2-DG treatment. Glucose consumption, ATP production, and lactate generation are significantly elevated by SFRP2 overexpression and reversed by 2-DG. *P < 0.05, **P < 0.01. (**G**) Cell proliferation assays using CCK-8 kit in HEY and SK-OV-3 cells transfected with NC, SFRP2, or SFRP2 + 2-DG. Overexpression of SFRP2 enhances proliferation, which is significantly blocked by 2-DG treatment. *P < 0.05, **P < 0.01, ***P < 0.001. (**H**) Colony formation assay showing long-term proliferative capacity in HEY and SK-OV-3 cells under the same conditions. SFRP2 overexpression increases colony number, which is abolished by co-treatment with 2-DG. ***P < 0.001

To elucidate the mechanism by which SFRP2 drives ovarian cancer progression, we performed GSEA on TCGA-OV transcriptomic data. Intriguingly, the C-C motif chemokine receptor 5 (CCR5) signaling pathway was the most significantly enriched gene set in the SFRP2 high-expression group (Fig. 3A). Given that CCR5 has been previously linked to tumor glycolysis [18], we hypothesized that SFRP2 might contribute to ovarian cancer progression by modulating aerobic glycolysis. To test this hypothesis, we first examined the effect of SFRP2 on key glycolytic indicators. Western blot analysis showed that knockdown of SFRP2 downregulated the expression of critical glycolytic enzymes, including PFKFB4 and LDHA (Fig. 3B). Functionally, SFRP2 knockdown led to a significant reduction in glucose consumption, lactate production, and intracellular ATP levels (Fig. 3C), all of which are hallmarks of glycolytic activity. Seahorse XF extracellular flux analysis further confirmed that SFRP2 depletion significantly decreased the ECAR (a measure of glycolytic flux) and increased the OCR (a measure of mitochondrial respiration) (Fig. 3D), indicating a metabolic shift away from glycolysis.

To ascertain whether glycolysis is essential for SFRP2-mediated oncogenic effects, we overexpressed SFRP2 and treated cells with the glycolytic inhibitor 2-Deoxy-D-glucose (2-DG). SFRP2 overexpression potently enhanced the expression of PKM2, HK2, GLUT1, ALDOC, ALDOA, ADH6 and LDHA (Fig. 3E), and increased glucose uptake, lactate and ATP production (Fig. 3F). These metabolic changes were accompanied by a promotion of cell proliferation and colony formation, key indicators of tumorigenic potential (Fig. 3G, H). Importantly, 2-DG treatment effectively abolished these pro-glycolytic and pro-tumorigenic effects induced by SFRP2 overexpression (Fig. 3E-H). These data conclusively demonstrate that SFRP2 facilitates ovarian cancer progression by augmenting aerobic glycolysis.

### SFRP2 promotes CEBPA-mediated transcriptional upregulation of PTK2B

**Fig. 4:**
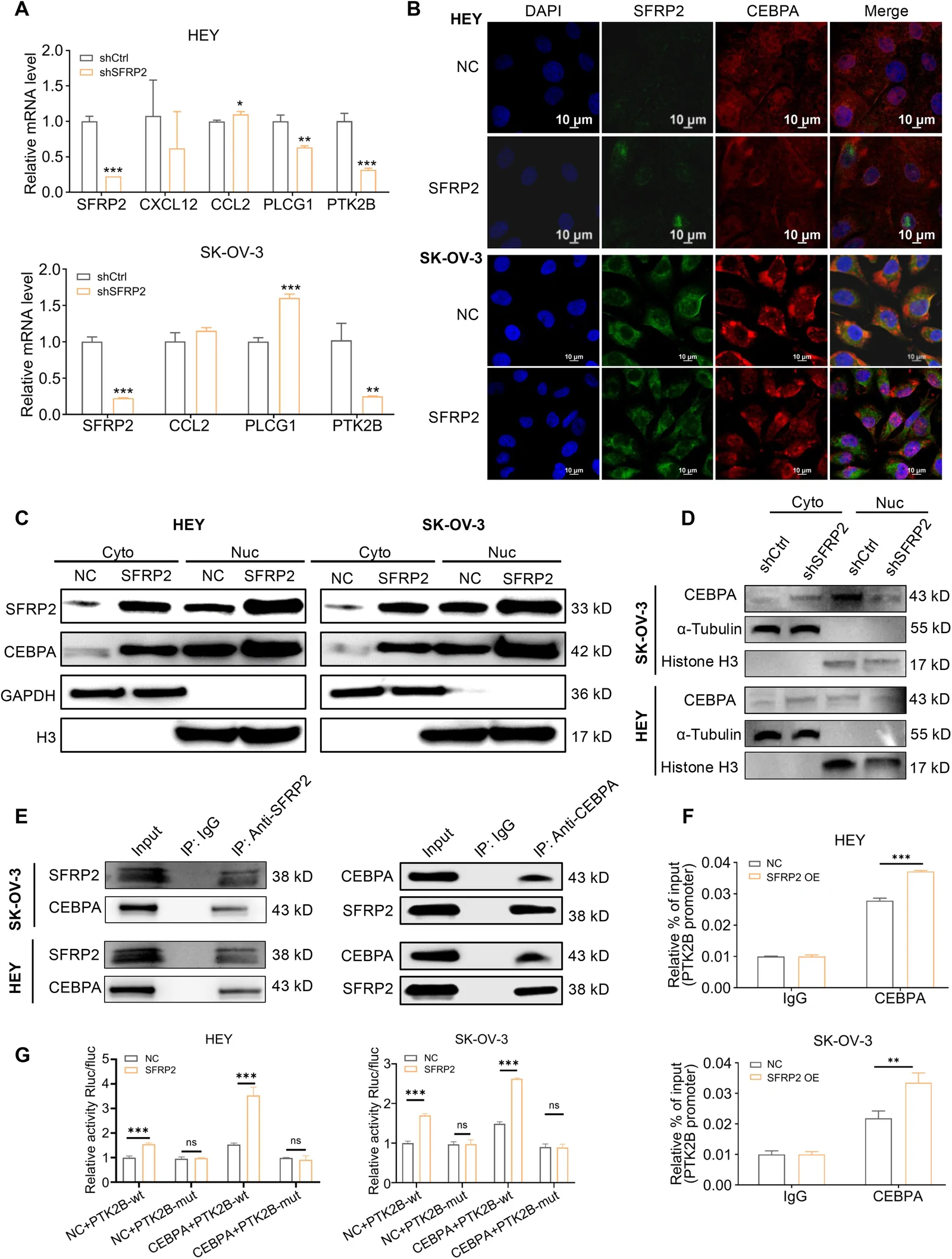
SFRP2 promotes CEBPA-mediated transcriptional upregulation of PTK2B. (**A**) Quantitative real-time PCR analysis of mRNA levels for SFRP2, CXCL12, CCL2, PLCG1, and PTK2B in HEY and SK-OV-3 cells following SFRP2 knockdown (shSFRP2) or control (shCtrl). Knockdown of SFRP2 significantly reduces PTK2B expression. *P < 0.05, **P < 0.01, ***P < 0.001. (**B**) Representative immunofluorescence (IF) images showing the subcellular localization and co-localization of SFRP2 (green) and CEBPA (red) in HEY and SK-OV-3 cells. Nuclei were stained with DAPI (blue). Scale bars = 10 μm. (**C**) Western blot analysis of SFRP2 and CEBPA protein levels in cytoplasmic and nuclear fractions of HEY and SK-OV-3 cells overexpressing SFRP2 (SFRP2-OE). GAPDH and Histone H3 were used as cytoplasmic and nuclear markers, respectively. (**D**) Western blot analysis of CEBPA protein levels in cytoplasmic and nuclear fractions after SFRP2 knockdown (shSFRP2) in SK-OV-3 and HEY cells. α-Tubulin and Histone H3 served as loading controls for the cytoplasm and nucleus, respectively. (**E**) Co-immunoprecipitation (Co-IP) assay demonstrating a direct physical interaction between endogenous SFRP2 and CEBPA proteins in SK-OV-3 and HEY cells. Cell lysates were immunoprecipitated with anti-SFRP2, anti-CEBPA antibody or IgG control, followed by Western blotting for CEBPA and SFRP2. (**F**) Chromatin immunoprecipitation (ChIP) assay assessing the enrichment of CEBPA on the PTK2B promoter region in HEY and SK-OV-3 cells overexpressing SFRP2 (SFRP2 OE) or control (NC). qPCR amplification of the PTK2B promoter region shows increased CEBPA binding in SFRP2-overexpressing cells compared to control. **P < 0.01, ***P < 0.001. (**G**) Dual-luciferase reporter assays evaluating the transcriptional activity of the PTK2B promoter. HEY and SK-OV-3 cells were co-transfected with wild-type (wt) or mutant (mut) PTK2B promoter luciferase constructs, along with SFRP2, CEBPA, or empty vector controls. SFRP2 overexpression enhances PTK2B promoter activity only when CEBPA is co-expressed, and this activation is abolished in the mutant promoter. ***P < 0.001

To identify the downstream effector mediating the oncogenic function of SFRP2, we focused on the CCR5 pathway-related genes. qPCR analysis revealed that knockdown of SFRP2 significantly reduced mRNA levels of PTK2B, but not other candidates, identifying PTK2B as a key potential downstream target (Fig. 4A). We next investigated how SFRP2 regulates PTK2B. As SFRP2 is a secreted protein, we hypothesized an indirect transcriptional mechanism. Using the STRING database to find SFRP2-interacting proteins and the hTFtarget database to predict transcription factors for the PTK2B promoter, we identified CEBPA as a common candidate (data not shown). To clarify the spatial context of the interaction between SFRP2 and CEBPA, we first investigated the subcellular localization of SFRP2. Immunofluorescence (IF) assays revealed a significant presence of SFRP2 in both the cytoplasm and the nucleus, where it notably co-localized with CEBPA in HEY and SK-OV-3 cells (Fig. 4B). To further validate this, we performed nucleocytoplasmic fractionation assays under both gain- and loss-of-function conditions. Western blot analysis showed that SFRP2 protein was distributed in both the cytoplasmic and nuclear compartments. Notably, SFRP2 overexpression markedly increased its nuclear abundance and simultaneously promoted the nuclear translocation of CEBPA (Fig. 4C). Conversely, knockdown of SFRP2 significantly reduced the nuclear accumulation of CEBPA (Fig. 4D). Consistent with these localization dynamics, Co-IP experiments confirmed a direct physical interaction between endogenous SFRP2 and CEBPA proteins in ovarian cancer cells (Fig. 4E). Importantly, to distinguish whether these effects depend on the extracellular/secreted pool or the intracellular pool of SFRP2, we performed intervention assays using an SFRP2-neutralizing antibody and recombinant SFRP2 protein. The results showed that neutralizing extracellular SFRP2 did not attenuate the SFRP2-induced glycolytic activity, nor did exogenous recombinant SFRP2 protein mimic these effects (Figure S2), firmly supporting that SFRP2 drives malignant progression through an intracellular rather than an autocrine mechanism. We then examined whether the SFRP2/CEBPA complex regulates PTK2B transcription. ChIP assays demonstrated that SFRP2 overexpression significantly enhanced the enrichment of CEBPA on the PTK2B promoter region (Fig. 4F). Consistent with this, dual-luciferase reporter assays showed that co-expression of SFRP2 and CEBPA synergistically activated the activity of a luciferase reporter driven by the PTK2B promoter (Fig. 4G). Taken together, these results delineate a novel mechanism whereby SFRP2 binds to CEBPA, promotes its nuclear translocation, and enhances its transcriptional activation of PTK2B.

### CEBPA serves as a critical functional mediator of the SFRP2-PTK2B axis

**Fig. 5:**
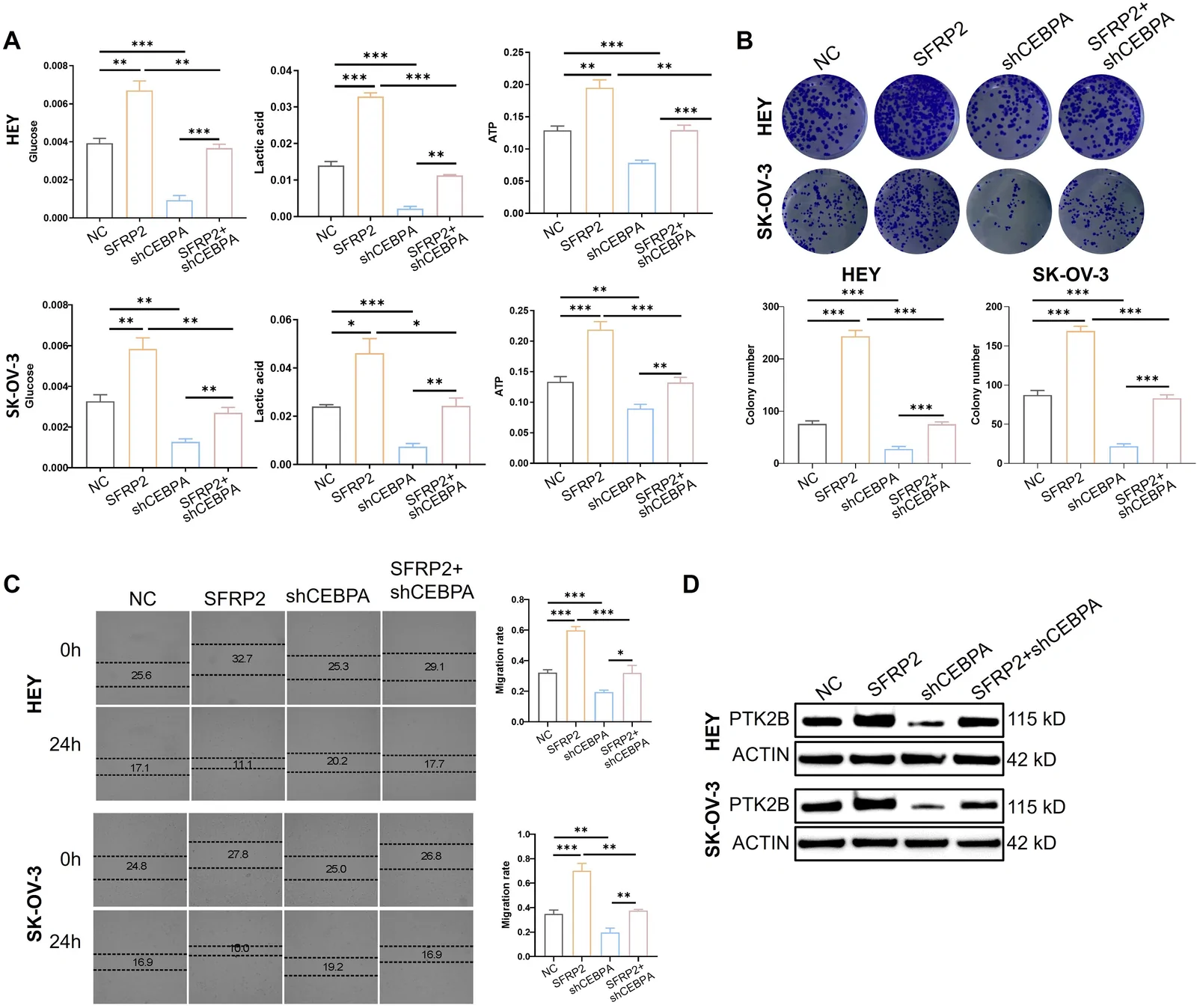
CEBPA is a critical functional mediator of SFRP2-driven aerobic glycolysis and malignant phenotypes in ovarian cancer. (**A**) Glycolytic activity was assessed by measuring glucose uptake (left), lactate production (middle), and intracellular ATP levels (right) in HEY and SK-OV-3 cells transfected with control vector (NC), SFRP2 overexpression (SFRP2), CEBPA knockdown (shCEBPA), or combined SFRP2 overexpression with CEBPA knockdown (SFRP2 + shCEBPA). *P < 0.05, **P < 0.01, ***P < 0.001. (**B**) Colony formation assays were performed to evaluate the proliferative capacity. Representative images (top) and quantitative analysis (bottom) are shown for both HEY and SK-OV-3 cells. ***P < 0.001. (**C**) Cell migration was examined using wound healing assays. Representative images at 0 h and 24 h (left) and quantification of migration rates (right) are presented. *P < 0.05, **P < 0.01, ***P < 0.001. (**D**) Western blot analysis of PTK2B protein expression in the indicated groups. ACTIN was used as a loading control

Given that SFRP2 directly interacts with and regulates the nuclear translocation of CEBPA, we first assessed the clinical relevance of CEBPA in ovarian cancer. Bioinformatic analysis using the GEPIA2 database revealed that CEBPA mRNA levels were significantly upregulated in ovarian cancer tissues (*n* = 426) compared with normal ovarian tissues (*n* = 88) (Figure S3), suggesting its potential oncogenic role. We next investigated whether the oncogenic effects of SFRP2 are dependent on CEBPA. Rescue experiments demonstrated that the promotion of glucose consumption, lactate production, and ATP generation induced by SFRP2 overexpression was significantly attenuated by CEBPA knockdown (Fig. 5A). Consistently, the enhanced colony formation and migratory velocity observed in SFRP2-overexpressing cells were largely reversed upon silencing CEBPA (Fig. 5B and C). Furthermore, the SFRP2-driven upregulation of PTK2B protein levels was markedly abolished following CEBPA depletion (Fig. 5D). These results indicate that CEBPA is an indispensable downstream effector that mediates SFRP2-induced metabolic reprogramming and malignant progression.

### The SFRP2/PTK2B axis promotes malignant progression through aerobic glycolysis

**Fig. 6:**
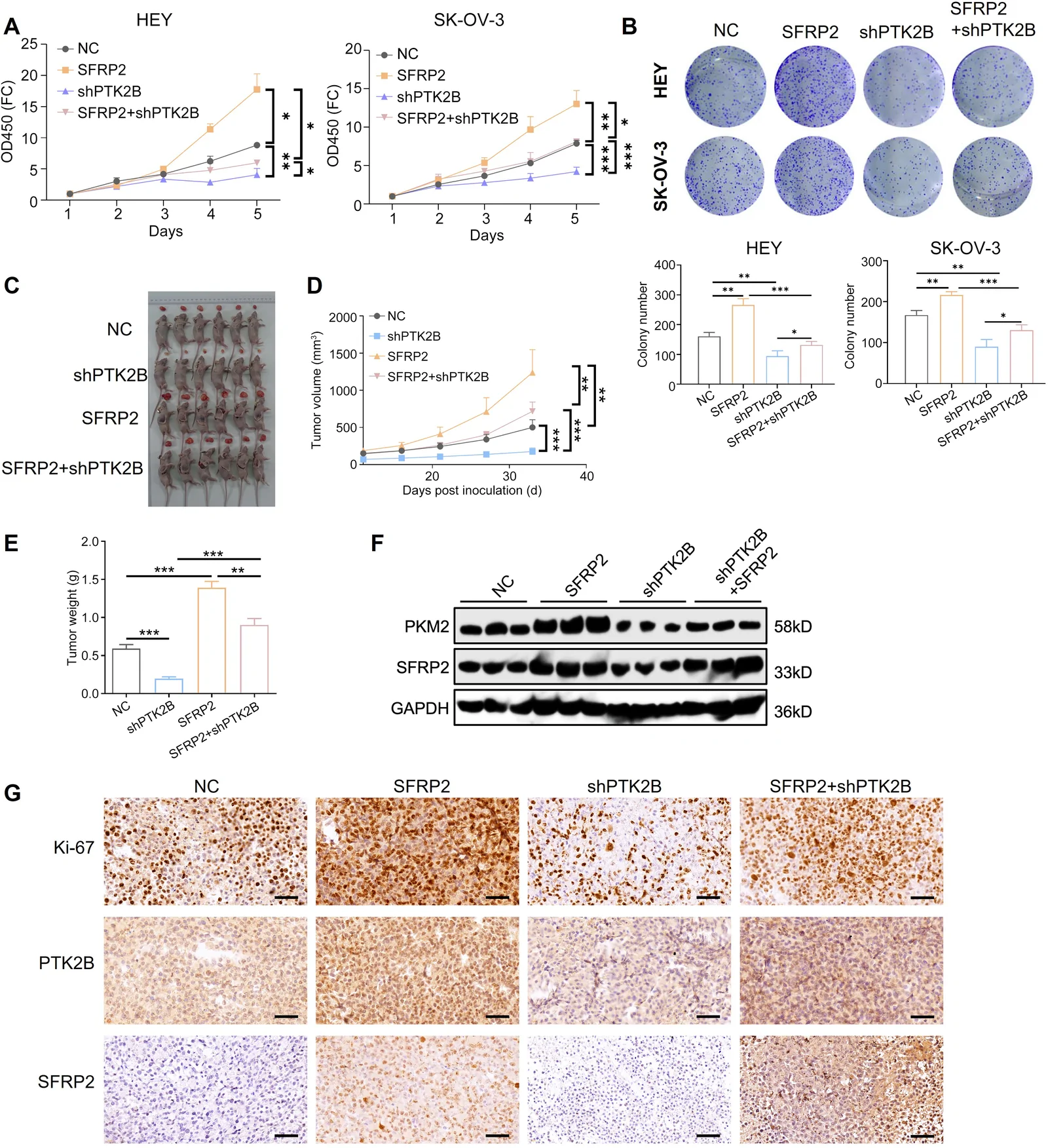
The SFRP2/PTK2B signaling axis promotes malignant progression through aerobic glycolysis. (**A**) CCK-8 showing growth kinetics of HEY and SK-OV-3 cells transfected with control (NC), SFRP2 overexpression (SFRP2), PTK2B knockdown (shPTK2B), or dual manipulation (SFRP2 + shPTK2B). SFRP2 overexpression significantly enhances cell proliferation, an effect reversed by co-knockdown of PTK2B. *P < 0.05, **P < 0.01, ***P < 0.001. (**B**) Colony formation assay demonstrating long-term proliferative capacity. Representative images and quantification show that SFRP2 overexpression increases colony number, which is abrogated by PTK2B knockdown. *P < 0.05, **P < 0.01, ***P < 0.001. (**C**) Representative image of subcutaneous xenograft tumors derived from HEY cells expressing NC, shPTK2B, SFRP2, or SFRP2 + shPTK2B after 33 days of inoculation in nude mice. (**D**) Tumor volume measured over time post-inoculation. SFRP2-overexpressing tumors grow significantly faster than controls, and this acceleration is blocked by PTK2B knockdown. **P < 0.01, ***P < 0.001. (**E**) Tumor weight at endpoint. SFRP2 overexpression results in significantly heavier tumors compared to control, and this effect is rescued by PTK2B knockdown. **P < 0.01, ***P < 0.001. (**F**) Western blot analysis of tumor lysates showing protein levels of SFRP2 and PKM2. SFRP2 overexpression upregulates PKM2 expression, an effect abolished by PTK2B knockdown. GAPDH serves as loading control. (**G**) IHC staining of tumor sections for Ki-67 (proliferation marker), PTK2B, and SFRP2. SFRP2 overexpression increases Ki-67 and PTK2B expression, while co-knockdown of PTK2B attenuates these signals. Scale bar = 50 μm

Finally, we conducted rescue experiments to confirm that PTK2B is the critical functional mediator of SFRP2. We established SFRP2-overexpressing HEY and SK-OV-3 cells, in which PTK2B was simultaneously knocked down (Figure S4). SFRP2 overexpression significantly promoted cell proliferation and colony formation, phenotypes that were effectively reversed upon PTK2B knockdown (Fig. 6A, B). These results indicate that PTK2B is indispensable for SFRP2-driven proliferative and clonogenic capacities.

We next extended these findings to an in vivo xenograft mouse model to assess the functional relevance of the SFRP2-PTK2B axis in tumor growth. Tumors derived from SFRP2-overexpressing cells exhibited significantly accelerated growth kinetics, resulting in markedly larger and heavier tumor masses compared to those derived from control cells. Strikingly, co-knockdown of PTK2B in SFRP2-overexpressing cells largely abolished these pro-tumorigenic effects, leading to markedly impaired tumor growth (Fig. 6C-E). Western blot analysis of tumor lysates showed that SFRP2 overexpression upregulated the glycolytic enzyme PKM2, and this effect was again abolished by PTK2B knockdown (Fig. 6F). Furthermore, IHC analysis of tumor sections revealed that SFRP2 overexpression increased the expression of PTK2B and the proliferation marker Ki-67, which was attenuated by PTK2B knockdown (Fig. 6G). These in vivo data robustly validate that SFRP2 exerts its pro-tumorigenic and pro-glycolytic effects primarily through PTK2B, underscoring the critical role of the SFRP2-PTK2B axis in driving ovarian cancer progression and metabolic reprogramming.

### PTK2B drives aerobic glycolysis via activating the AKT/mTOR signaling pathway

**Fig. 7:**
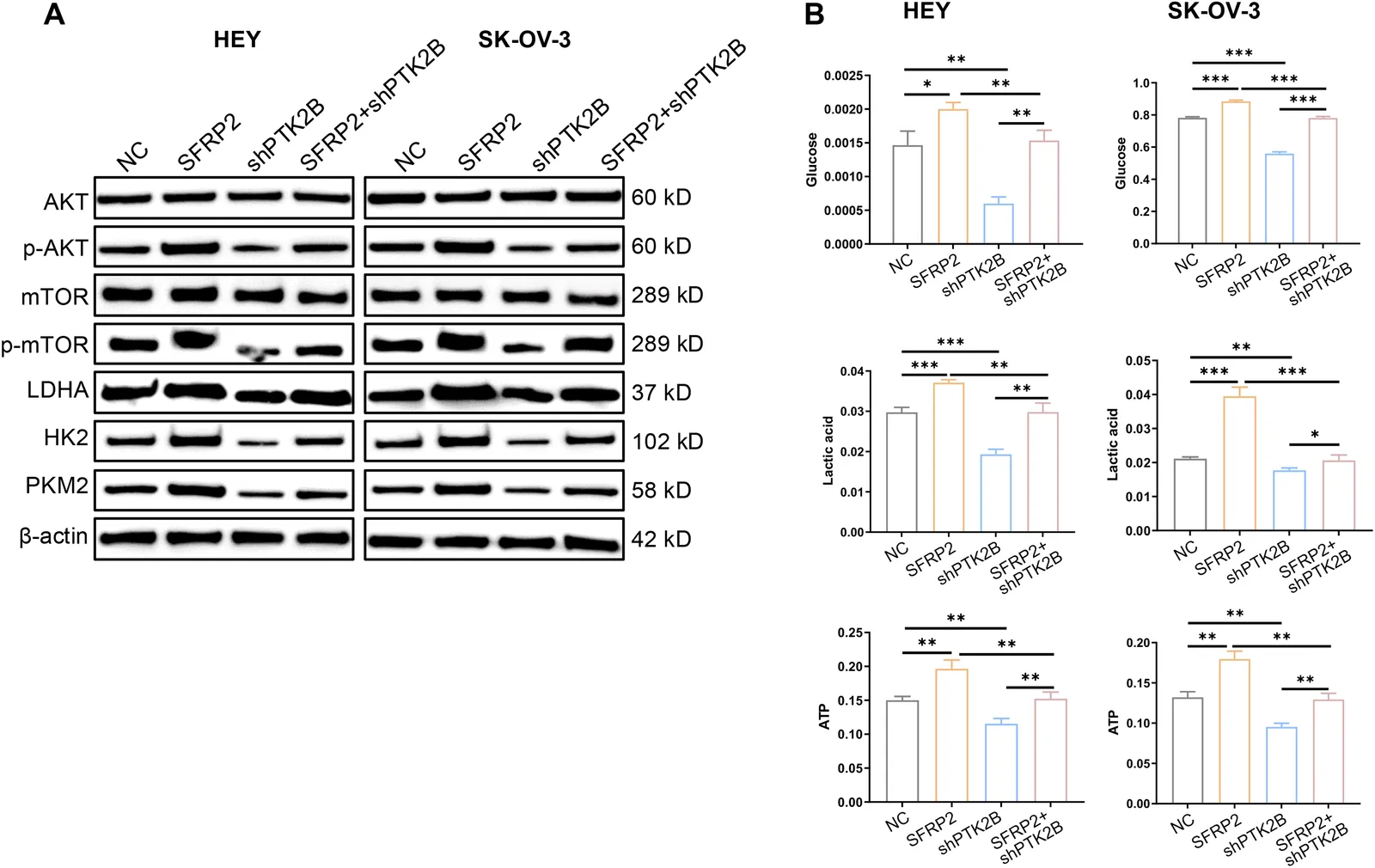
PTK2B drives SFRP2-mediated aerobic glycolysis via activation of the AKT/mTOR signaling pathway. (**A**) Western blot analysis of AKT, p-AKT, mTOR, p-mTOR, and glycolytic enzymes (LDHA, HK2, PKM2) in HEY and SK-OV-3 cells transfected with NC, SFRP2-OE, shPTK2B, or SFRP2-OE+shPTK2B. β-actin was used as the loading control. (**B**) Measurement of glucose uptake, lactate production, and intracellular ATP levels in HEY and SK-OV-3 cells from the indicated groups. *P < 0.05, **P < 0.01, ***P < 0.001

To further elucidate the molecular mechanism by which PTK2B regulates aerobic glycolysis, we investigated the downstream phospho-signaling events. Given that the PI3K-AKT-mTOR cascade is a well-established master regulator of glucose metabolism, we examined this pathway in our rescue model. Western blot analysis revealed that SFRP2 overexpression significantly increased the phosphorylation levels of AKT and mTOR, accompanied by the upregulation of key glycolytic enzymes including HK2, PKM2, and LDHA in both HEY and SK-OV-3 cells. Notably, these effects were markedly reversed by PTK2B knockdown, whereas total AKT and mTOR protein levels remained unaffected (Fig. 7A). Consistently, the SFRP2-induced enhancement in glucose consumption, lactate production, and ATP generation was effectively abolished upon PTK2B depletion (Fig. 7B). Together, these results indicate that PTK2B drives SFRP2-mediated aerobic glycolysis through activation of the AKT/mTOR signaling pathway and subsequent upregulation of glycolytic enzymes.

## Discussion

This study identifies a new oncogenic pathway—SFRP2/CEBPA/PTK2B—that drives malignant progression by activating aerobic glycolysis. We provide evidence that SFRP2, beyond its canonical role as a secreted antagonist, exerts a non-canonical intracellular function by directly binding CEBPA, facilitating its nuclear translocation and co-activating PTK2B expression. Importantly, we demonstrated that this effect is driven by the intracellular pool of SFRP2, as extracellular interventions using neutralizing antibodies or recombinant protein failed to modulate glycolytic phenotypes. The subsequent upregulation of PTK2B is indispensable for glycolytic flux and aggressive tumor phenotypes driven by SFRP2, as validated in vitro and in vivo.

SFRP2 is a key regulator of the Wnt signaling pathway and plays complex roles in various cancers. In colorectal cancer, SFRP2 activates enolase 2 (ENO2) through the TCF4/β-catenin axis, significantly promoting glycolysis and metastatic phenotypes. Inhibition of ENO2 reverses SFRP2-induced metastasis, and co-expression of SFRP2/ENO2 is associated with poorer survival and higher recurrence rates [19]. In non-small cell lung cancer, m6A-methylated SFRP2 is recognized and interacts with YTHDF2; knockdown of SFRP2 suppresses glycolysis and cancer stem cell properties [20]. Current research on SFRP2 in ovarian cancer still has several gaps, such as its interaction with known glycolytic regulators (e.g., HIF-1α, FOXK2 [21, 22]) and its subtype-specific roles in different ovarian cancer subtypes [23]. Existing evidence suggests that targeting SFRP2 and its downstream glycolytic pathways may offer new strategies for improving ovarian cancer prognosis [19, 24]. Our findings robustly position SFRP2 as an oncogene in ovarian cancer, consistently demonstrating that its high expression correlates with advanced disease and poor survival. We extend the understanding of SFRP2’s oncogenic functions by establishing its critical role in promoting proliferation, clonogenicity, migration, and anti-apoptosis in ovarian cancer cells. More importantly, we uncover a previously unrecognized function of SFRP2 in regulating cancer cell metabolism. While a recent study suggested SFRP2’s involvement in metabolism in other contexts [19], its direct role in driving aerobic glycolysis, the Warburg effect, in ovarian cancer is a novel finding. Our data demonstrate that SFRP2 significantly enhances glycolytic flux, and this metabolic reprogramming is essential for its tumor-promoting effects, as inhibition of glycolysis abrogated SFRP2-driven oncogenic phenotypes.

Bioinformatic GSEA of the TCGA-OV dataset revealed enrichment of the CCR5 signaling pathway in SFRP2-high tumors. Treating this finding strictly as a hypothesis-generating lead rather than a mechanistic link, we screened downstream candidates within this gene set by qPCR and identified PTK2B as the most significantly downregulated target upon SFRP2 knockdown [25]. PTK2B is a non-receptor tyrosine kinase known for its roles in cell adhesion, migration, and survival in various cancers [26–28]. Emerging evidence has begun to link PTK2B to metabolic regulation. For instance, the interaction between PTK2B and SIRPα in macrophages regulates both autophagy and necroptosis, processes closely linked to cellular metabolic reprogramming such as glycolysis and mitochondrial function, further supporting the role of PTK2B in immune-metabolic crosstalk [29]. However, its role in ovarian cancer metabolism, particularly in glycolysis, remained unexplored. Our study clearly demonstrates that PTK2B is a critical mediator of SFRP2-induced aerobic glycolysis and tumor progression in ovarian cancer. Knockdown of PTK2B reversed the pro-glycolytic and pro-tumorigenic effects of SFRP2 both in vitro and in vivo. This aligns with and extends the emerging concept of PTK2B as a metabolic regulator in cancer, providing a direct link between SFRP2 signaling and PTK2B-dependent glycolytic reprogramming.

The transcriptional mechanism delineated here is particularly intriguing. SFRP2, typically considered a secreted Wnt antagonist, is shown to have an intracellular role by directly interacting with CEBPA and influencing its subcellular localization and transcriptional activity. Through nucleocytoplasmic fractionation and immunofluorescence, we confirmed that SFRP2 protein itself localizes to the nucleus, where it forms a complex with the transcription factor CEBPA. Our rescue experiments robustly demonstrated that CEBPA is functionally indispensable for SFRP2-mediated glycolytic and oncogenic effects. CEBPA is a transcription factor involved in differentiation and metabolism [30]. Our ChIP and luciferase reporter assays confirm that the SFRP2/CEBPA complex directly binds to and activates the PTK2B promoter, establishing a linear pathway from SFRP2 to PTK2B upregulation. This non-canonical, intracellular function expands the paradigm of SFRP2’s actions in cancer and suggests potential novel therapeutic strategies aimed at disrupting this protein-protein interaction.

In summary, our work delineates a previously unrecognized SFRP2/CEBPA/PTK2B axis that is critical for aerobic glycolysis and malignant progression in ovarian cancer. These findings not only deepen our understanding of the molecular mechanisms driving metabolic reprogramming in this deadly disease but also highlight SFRP2 and PTK2B as promising prognostic biomarkers and attractive therapeutic targets. Targeting this axis, particularly through strategies focusing on its intracellular interactions or downstream signaling, could represent a novel metabolic therapy for ovarian cancer patients.

## Supplementary Information

Below is the link to the electronic supplementary material.

Supplementary Material 1

Supplementary Material 2

Supplementary Material 3

Supplementary Material 4

Supplementary Material 5: Supplementary Figure 1. Validation of SFRP2 knockdown in ovarian cancer cells. (A) Relative SFRP2 mRNA levels in HEY cells transfected with control (CON), shCtrl, or three distinct shRNA constructs targeting SFRP2 (shSFRP2-1, -2, -3). qRT-PCR shows significant reduction in SFRP2 expression by shSFRP2-1 and shSFRP2-3 compared to control. **P < 0.01, ***P < 0.001. (B) Relative SFRP2 mRNA levels in HEY and SK-OV-3 cells transfected with shCtrl, shSFRP2-1, or shSFRP2-3. Both shSFRP2-1 and shSFRP2-3 effectively reduce SFRP2 expression in both cell lines. *P < 0.05, ***P < 0.001. (C) Western blot analysis of SFRP2 protein levels in HEY and SK-OV-3 cells after transfection with shCtrl, shSFRP2-1, or shSFRP2-3. GAPDH serves as loading control. Both shSFRP2-1 and shSFRP2-3 significantly reduce SFRP2 protein expression, confirming efficient knockdown at the protein level.

Supplementary Material 6: Supplementary Figure 2. CEBPA is significantly upregulated in ovarian cancer tissues. Bioinformatic analysis of CEBPA mRNA expression in ovarian cancer (OV) tissues (n=426, red) compared with normal ovarian tissues (n=88, gray) was performed using the GEPIA2 online database (http://gepia2.cancer-pku.cn/), which integrates RNA sequencing data from the TCGA and GTEx projects. Data are presented as log₂(TPM+1). *P < 0.05 versus normal tissues.

Supplementary Material 7: Supplementary Figure 3. Validation of rescue models in ovarian cancer cells. (A) Relative SFRP2 and PTK2B mRNA levels in HEY and SK-OV-3 cells following SFRP2 overexpression (SFRP2 vs. NC) or PTK2B knockdown (shPTK2B vs. shCtrl). SFRP2 overexpression increases SFRP2 mRNA levels, while shPTK2B efficiently reduces PTK2B expression. GAPDH serves as loading control. **P < 0.01, ***P < 0.001. (B) Western blot analysis of SFRP2 and PTK2B protein levels in HEY and SK-OV-3 cells following indicated plasmids transfection. GAPDH serves as loading control.

Supplementary Material 8: Supplementary Figure 4. The pro-glycolytic effect of SFRP2 is mediated by its intracellular pool rather than extracellular/secreted SFRP2. Lactic acid production was measured in HEY and SK-OV-3 cells under the following conditions: control (Con), SFRP2 overexpression (SFRP2), SFRP2-overexpressing cells treated with an SFRP2-neutralizing antibody (SFRP2+anti-SFRP2 Ab, 2 μg/mL), and control cells treated with exogenous recombinant human SFRP2 protein (Con+Recombinant SFRP2, 500 ng/mL). ***P < 0.001; ns, non-significant.

## Data Availability

The data that support the findings of this study are available from the corresponding author upon reasonable request.
