# Supplementary material for: SFRP2 drives aerobic glycolysis and tumor progression in ovarian cancer by transcriptional upregulation of PTK2B
Source: J Transl Med. 2026 Jun 23;24:1153. doi: 10.1186/s12967-026-08480-9 (PMC13551736; doi:10.1186/s12967-026-08480-9)
Supplement: Supplementary file 1 [file 12967_2026_8480_MOESM1_ESM.docx]

**Table S1** Target sequences.

| Gene | | No. | Target sequence (5'-3') |
| --- | --- | --- | --- |
| SFRP2 | SFRP2-1 | | ACCGAGGAAGCTCCAAAGGTA |
| SFRP2 | SFRP2-2 | | GTCCGAAAGGGACCTGAAGAA |
| SFRP2 | SFRP2-3 | | GCAGAGAGAGTTCAAGCGCAT |
| PTK2B | PTK2B-1 | | TGCCATGGAGCAAGAGAGGAA |
| PTK2B | PTK2B-2 | | CCTAGCAGAGGCTGAGAACAT |
| PTK2B | PTK2B-3 | | CGAGGCAGTGATCATGAAGAA |
