# Supplementary material for: SFRP2 drives aerobic glycolysis and tumor progression in ovarian cancer by transcriptional upregulation of PTK2B
Source: J Transl Med. 2026 Jun 23;24:1153. doi: 10.1186/s12967-026-08480-9 (PMC13551736; doi:10.1186/s12967-026-08480-9)
Supplement: Supplementary file 2 [file 12967_2026_8480_MOESM2_ESM.docx]

**Table S2** Primers used in qPCR and ChIP-qPCR.

| Gene | Forward primer sequence (5’-3’) | Reverse primer sequence (5’-3’) |
| --- | --- | --- |
| SFRP2 | CCACCGAGGAAGCTCCAAA | GGACACACCGTTCAGCTTGT |
| CXCL12 | ATTCTCAACACTCCAAACTGTGC | ACTTTAGCTTCGGGTCAATGC |
| CCL2 | AGAATCACCAGCAGCAAG | GGAATCCTGAACCCACTT |
| PLCG1 | GCCTATGCAGATGAACCAGG | CATTCTTTGGCAGATGTCGG |
| PTK2B | TCCGGTTGGCTGAGTGCTATG | CACCTCACCCACCGTCATCTGT |
| PTK2B-promoter | AGCTCCTCCTCTCCAGTCCAT | CCTCCTCTGCCTCCCAAACTAC |
| GAPDH | TGACTTCAACAGCGACACCCA | CACCCTGTTGCTGTAGCCAAA |
