# Supplementary material for: SFRP2 drives aerobic glycolysis and tumor progression in ovarian cancer by transcriptional upregulation of PTK2B
Source: J Transl Med. 2026 Jun 23;24:1153. doi: 10.1186/s12967-026-08480-9 (PMC13551736; doi:10.1186/s12967-026-08480-9)
Supplement: Supplementary file 3 [file 12967_2026_8480_MOESM3_ESM.docx]

**Table S3** Antibodies used in WB.

| Primary antibodies | Dilution in WB | Source species | Company | Catalog No. |
| --- | --- | --- | --- | --- |
| SFRP2 | 1:2000 | Rabbit | Proteintech | 66328-1-lg |
| PTK2B | 1:5000 | Mouse | Proteintech | 67141-1-lg |
| PKM2 | 1:5000 | Rabbit | Proteintech | 15822-1-AP |
| HK2 | 1:3000 | Mouse | Proteintech | 66974-1-lg |
| GLUT1 | 1:1000 | Rabbit | Proteintech | 21829-1-AP |
| ADH6 | 1:5000 | Mouse | Proteintech | 67709-1-lg |
| ALDOA | 1:3000 | Rabbit | Proteintech | 11217-1-AP |
| ALDOC | 1:5000 | Rabbit | Proteintech | 14884-1-AP |
| LDHA | 1:3000 | Rabbit | Abcam | Ab52488 |
| β-ACTIN | 1:4000 | Mouse | Proteintech | 66009-1-lg |
| GAPDH | 1:30000 | Mouse | Proteintech | 60004-1-lg |
