# Supplementary material for: SFRP2 drives aerobic glycolysis and tumor progression in ovarian cancer by transcriptional upregulation of PTK2B
Source: J Transl Med. 2026 Jun 23;24:1153. doi: 10.1186/s12967-026-08480-9 (PMC13551736; doi:10.1186/s12967-026-08480-9)
Supplement: Supplementary file 4 [file 12967_2026_8480_MOESM4_ESM.docx]

**Table S4.** Univariate and multivariate Cox proportional hazards analysis for overall survival in patients with ovarian cancer.

| **Variables** | **Univariate Analysis** |  | **Multivariate Analysis** |  |
| --- | --- | --- | --- | --- |
|  | **HR (95% CI)** | ***P* value** | **HR (95% CI)** | ***P* value** |
| SFRP2 expression (High vs. Low) | 0.923 (0.472–1.804) | 0.814 | 1.072 (0.474–2.426) | 0.868 |
| Pathological grade | - | - | 0.148 (0.027–0.795) | 0.026* |
| Tumor size (≥ 5 cm vs. < 5 cm) | - | - | 1.245 (0.534–2.902) | 0.612 |
| Age (≥ 53 vs. < 53 years) | - | - | 1.259 (0.593–2.672) | 0.549 |
| FIGO stage | - | - | 1.180 (0.706–1.975) | 0.528 |

Notes: HR, hazard ratio; CI, confidence interval; FIGO, International Federation of Gynecology and Obstetrics.
*P < 0.05 indicates statistical significance.
